# High *Plasmodium* infection and multiple insecticide resistance in a major malaria vector *Anopheles coluzzii* from Sahel of Niger Republic

**DOI:** 10.1186/s12936-019-2812-0

**Published:** 2019-05-24

**Authors:** Sulaiman S. Ibrahim, Muhammad M. Mukhtar, Helen Irving, Rabiou Labbo, Michael O. Kusimo, Izamné Mahamadou, Charles S. Wondji

**Affiliations:** 1 Vector Biology Department, Liverpool School of Tropical Medicine (LSTM), Pembroke Place, L3 5QA UK; 2 Department of Biochemistry, Bayero University, PMB 3011, Kano, Nigeria; 3 Centre de Recherche Médicale et Sanitaire (CERMES), Institut Pasteur International Network, 634 Bd de la Nation, BP 10887, Niamey, Niger; 4 LSTM Research Unit, Centre for Research in Infectious Diseases (CRID), P.O. Box 13591, Yaoundé, Cameroon

**Keywords:** *Plasmodium falciparum*, *An. coluzzii*, Malaria, Sahel, Insecticides, Metabolic, Resistance, *kdr*

## Abstract

**Background:**

Information on insecticide resistance and the mechanisms driving it in the major malaria vectors is grossly lacking in Niger Republic, thus hindering control efforts. To facilitate evidence-based malaria control, the role of *Anopheles coluzzii* population from southern Niger, in malaria transmission, its insecticides resistance profile and the molecular mechanisms driving the resistance were characterized.

**Methods:**

Blood fed female *Anopheles gambiae* sensu lato resting indoor were collected at Tessaoua, Niger. Source of blood was established using PCR and infection with *Plasmodium* determined using TaqMan assay. Resistance profile was established with the major public health insecticides, and resistance intensity determined with deltamethrin. Synergist assays were conducted with piperonyl butoxide and diethyl maleate. Presence of L1014F and L1014S knockdown resistance (*kdr*) mutations in the voltage-gated sodium channel (VGSC) was investigated using TaqMan genotyping, and strength of selection pressure acting on the *Anopheles* populations determined by assessing the genetic diversity of a fragment spanning exon-20 of the VGSC from alive and dead females.

**Results:**

High human blood index (96%) and high *Plasmodium falciparum* infection (~ 13%) was observed in the *An. coluzzii* population. Also, a single mosquito was found infected with *Plasmodium vivax.* High pyrethroid and organochloride resistance was observed with mortalities of less than 20% for deltamethrin, permethrin, α-cypermethrin, and DDT. A high LD_50_ (156.65 min) was obtained for deltamethrin, with a resistance ratio of ~ 47.18 compared to the susceptible Ngoussou colony. Moderate carbamate resistance was observed, and a full susceptibility to organophosphates recorded. Synergist bioassays with piperonyl butoxide and diethyl maleate significantly recovered deltamethrin and DDT susceptibility, respectively implicating CYP450 s (mortality = 82%, χ^2^ = 84.51, p < 0.0001) and glutathione S-transferases (mortality = 58%, χ^2^ = 33.96, p < 0.001) in resistance. A high frequency of 1014F *kdr* mutation (82%) was established, with significant difference in genotype distribution associated with permethrin resistance [odds ratio = 7.71 (95% CI 2.43–14.53, χ^2^ = 13.67, p = 0.001]. Sequencing of intron-1 of the voltage-gated sodium channel (VGSC) revealed a low genetic diversity.

**Conclusion:**

High pyrethroid resistance highlight the challenges to the effectiveness of the pyrethroids-based ITNs and indoor residual spraying (IRS) against *An. coluzzii* in Niger. The pyrethroids-synergists LLINs and organophosphate-based IRS maybe the alternatives for malaria control in southern Niger.

## Background

The global fight against malaria is at a crossroads with the progress made for almost two decades stalled [1, 2]. The decline in malaria burden between 2000 and 2015 [3] is facing serious setbacks, as cases of malaria have consistently increased globally between 2015 and 2017 [1, 4]. This malaria transmission recurrence, threatening a return to the 2012 levels is a warning sign to the control and elimination efforts of the World Health Organization (WHO) [5]. The trend suggests that primary regions of interests for pre-elimination need urgent attention; most worrying being the WHO African Region, which accounts for ~ 92% of all malaria-related deaths [2]. Niger Republic, with a high transmission and increased case incidence (2010–2016), is among countries with the highest per capita rate of malaria fatalities globally [6]. It alone accounts for ~ 4% of malaria burden in the world [4]. The Sahelian region of Niger represents the northern limit of malaria endemicity, compared to the north of the country where malaria is more marginal, where population densities are lower, and *Anopheles* densities are known to be very low [7]. The Sahel Region, characterized by a high seasonal malaria transmission [8], is important for monitoring purposes, to provide enough evidences to support elimination in West Africa. However, generating reliable data on indigenous Sahelian *Anopheles* species, their contribution to malaria transmission and monitoring insecticide resistance status over time and space [4], is a pre-requisite for implementation of evidence-based control measures in this dry region.

The major vectors of malaria in Niger are *Anopheles gambiae* sensu lato (s.l.) and *Anopheles funestus* [4, 9, 10], with *Anopheles coluzzii* being the dominant species from the Gambiae complex [11]. Unfortunately, information on the insecticide resistance status of these dominant vector species (DVS) from Niger and the underlying molecular mechanisms driving the resistance in the field is very limited. In addition, there is also a dearth of information on the impact of nationwide distribution of long-lasting insecticidal bed nets on development and/or escalation of resistance. Between 2005 and 2009 more than 6 million LLINs (Permanet ^®^2.0, Vestergaard, Lausanne, Switzerland) were freely distributed, with 73.4% coverage. Additionally, 11.3 million nets were distributed between 2014 and 2017 [12]. A study published in 2017 described pyrethroids and DDT resistance in *An. coluzzii* from Niger [9]. However, collection for the study was carried out in 2013 and the link between the presence of the observed 1014F *kdr* mutation and the resistance phenotype was not established. Another notable study followed increased frequency of 1014F *kdr* in *An. coluzzii* following the nationwide mass distribution of bed nets in Niger [7]. However, pyrethroids/DDT resistance profiling was not conducted on the *Anopheles* used in the study prior to genotyping.

To facilitate planning and implementation of evidence-based malaria control in Niger/the Sahel, the role of a major malaria vector *An. coluzzii*, in malaria transmission in Niger, and its resistance status to the various insecticides in use for public health control was characterized. The underlying molecular mechanisms driving the resistance in the field was also investigated.

## Methods

### Study site and collection

**Fig. 1:**
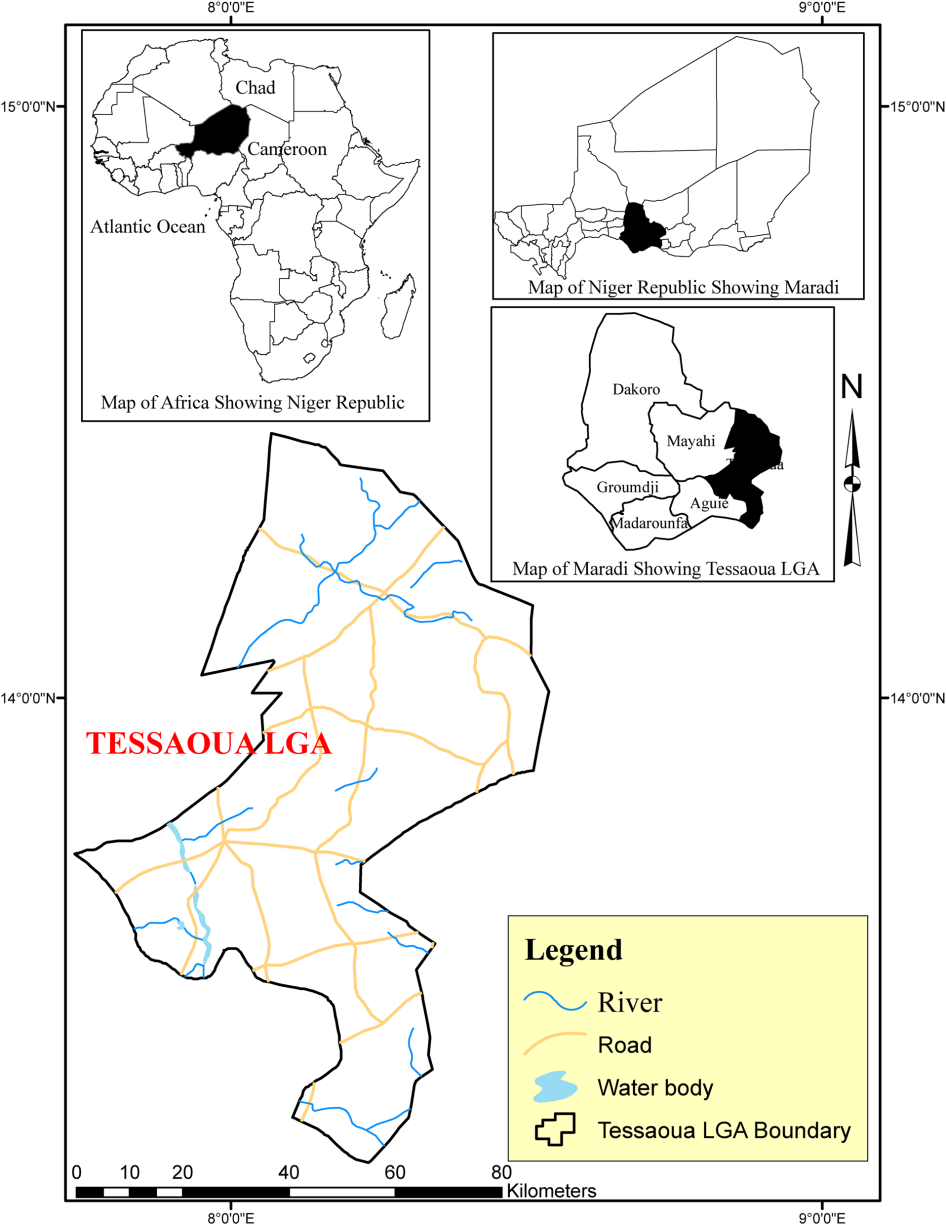
A map showing the field collection site in the Sahel of Tessaoua, Niger Republic

Clearance for field work was sought and granted by the Niger Federal Ministry of Public Health (Authorization Number 003210/MSP/SG/DEP/DER). Indoor-resting mosquitoes were collected using battery-operated aspirators (John. W. Hock, Florida, USA) from six randomly selected houses, in the early morning hours (5:30 a.m.–6:30), in Takatsaba village, Tessaoua Department (13.7559°N, 7.9865°E), Maradi State, Niger (Fig. 1). Collection was conducted every morning for 3 days (12–14 of September 2017). Takatsaba is a farming community, which banked the Goulbi River, allowing extended cultivation of rice and vegetables. Blood fed females were maintained on 10% sugar at 25 °C ± 2 and 70–75% relative humidity until fully gravid. 316 blood fed *An. coluzzii* females were individually transferred into 1.5 ml tubes and forced to lay eggs, using established protocol [13]. The F_0_ parents identified as belonging to *An. gambiae* complex using morphological keys [14] and confirmed as *An. coluzzii* using the SINE200-PCR [15] were selected for egg laying. Male *Anopheles* and *Culex* mosquitoes were discarded. Egg batches were transferred into paper cups for hatching in the insectary at Bayero University, Kano, Nigeria. Hatched eggs were pooled into larvae bowls and supplemented with Tetramin™ baby fish food. 2- to 4-days old F_1_ female adults that emerged were randomly mixed in cages and used for insecticide bioassays.

To establish indoor resting densities of the *Anopheles*, infection with *Plasmodium* and source of blood meal, pyrethrum spray collection (PSC) was also conducted on the 4th day (15 of September 2017), following protocols as advised by the WHO [16]. PSC was carried out in 8 randomly selected houses in early morning hours (6:00 a.m.–7:00 a.m.). Mosquitoes were sorted according to morphological keys as either belonging to *An. gambiae* complex [14] or as *Culex* species and stored individually in PCR strips with silica gel.

### Anopheles species identification

Genomic DNA was extracted from all the female *Anopheles* mosquitoes which successfully laid eggs using the Livak protocol [17]. Species identification to the molecular level was carried out using the SINE200 PCR [15]. All female *An. gambiae* sensu lato collected from PSC were dissected to separate heads/thoraces from the abdomens. DNA was extracted from these heads/thoraces and species identification carried out as described above. These DNA samples were transported to the Liverpool School of Tropical Medicine (LSTM), UK, under the DEFRA license (PATH/125/2012,) for downstream molecular analyses.

### Estimation of transmission parameters

#### Indoor resting density and human blood index estimation

The *Anopheles* indoor resting density (IRD) was calculated from the number of female *Anopheles* caught using the PSC in relation to the number of houses sampled as advised by the WHO [16, 18].

DNA was extracted from the abdomen of all blood fed female *Anopheles* as advised in the WHO guidelines [16]. This was done using the DNeasy Blood and Tissue Kit (QIAGEN, Hilden, Germany) according to manufacturer’s protocol. The human blood index was established from the proportion of blood fed *Anopheles* that have fed on humans relative to the total number of blood fed *Anopheles* caught, following a cocktail PCR of Kent and Norris et al. [19].

#### Sporozoite infection rate

DNA from 93 female *An. coluzzii* collected using PSC were used to detect sporozoite using a TaqMan genotyping assay [20]. Mosquitoes were stored immediately after collection in a silica gel and DNA extracted within 7 days. To minimize blood contamination heads/thoraces were separated from abdomens at the anterior end of the abdomen [21]. Real-time PCR MX 3005 (Agilent, Santa Clara, USA) was used for the amplification. 1 μl of gDNA extracted from each head/thorax was used, with an initial denaturation at 95 °C for 10 min, followed by 40 cycles each of 15 s at 95 °C and 1 min at 60 °C. Primers described by Bass [20] were used together with two probes labelled with fluorophores, FAM to detect *Plasmodium falciparum*, and HEX to detect combination of *Plasmodium ovale, Plasmodium vivax* and *Plasmodium malariae*. Positive controls (known FAM+ and OVM +) were used, in addition to a negative control (1 μl of ddH_2_0). TaqMan assay results were validated using a nested PCR [22]. This was carried out using all the positive TaqMan samples. Sporozoite rate was calculated as percentage of mosquitoes with sporozoites relative to the total number of the females examined [16].

### Tests for insecticides susceptibility

Insecticide susceptibility bioassays were performed according to the WHO protocol [23] with the insecticides from four major public health classes. Four replicates of F_1_ (20–25, 2–4 days old females) per tube were used for each insecticide, alongside 25 unexposed females (control). To confirm the efficacy of the papers, the fully susceptible *An. coluzzii* (Ngoussou colony) [24] was tested first or simultaneously with the experimental populations. Eight (8) insecticides were tested, including: (i) the type I pyrethroid: permethrin (0.75%); (ii) the type II pyrethroids: deltamethrin (0.05%) and α-cypermethrin (0.05%); (iii) the organochlorine: DDT (4%); (iv) the carbamates: bendiocarb (0.1%) and propoxur (0.1%); and (v) the organophosphates: malathion (5%) and pirimiphos-methyl (0.25%). Knockdown rates were recorded for permethrin, deltamethrin and DDT during the exposure, at intervals of 15 min, 30 min, 45 min and 1 h. After 1 h exposure mosquitoes were transferred to holding tubes and supplied with 10% sugar. Mortality was recorded 24 h after exposure. Populations were considered susceptible to an insecticide where mortality was > 98%, suspected to be resistant (moderately resistant) where mortality is between 90 and 98%, and resistant where mortality was found to be < 90% [25].

### Estimation of resistance intensity with time-course bioassays

To establish the strength of pyrethroid resistance with time, additional bioassays were performed with 0.05% deltamethrin. 20–25 F_1_ females in 4 replicates were exposed in time-course bioassays varying exposure times from 60 to 300 min. Protocols were as described above in conventional bioassays, except for variation in time. For the fully susceptible Ngoussou LT_50_ have been established previously with discriminating concentration of deltamethrin in time-course bioassays spanning 2.5 to 60 min (Ibrahim et al., In Press, http://www.nature.com/articles/s41598-019-43634-4). Resistance intensity was established by comparing the LT_50_ calculated for the Takatsaba *An. coluzzii* to that of the Ngoussou colony.

### Synergist bioassays

To establish the potential role of detoxification enzyme systems in the observed resistance, synergist bioassays were conducted with 2–4 days old F_1_ females, with 4% piperonyl butoxide (PBO: an inhibitor of CYP450s [26]) against deltamethrin, and 8% diethyl maleate (DEM: an inhibitor glutathione S-transferases [27]). Initially, 20–25 females were pre-exposed to a synergist for 1 h and then transferred to tubes containing deltamethrin for 1 h [25]. Mosquitoes were treated as in the bioassays described above and mortalities scored after 24 h. Two controls were used: (i) 25 females exposed to only control paper (with neither a synergist, nor any insecticide); and (ii) 25 females each exposed to either PBO or DEM only.

### Polymorphism analysis of the voltage-gated sodium channel

#### Genotyping of L1014F and L1014S kdr mutations

To assess the frequency and relation between *kdr* genotypes and pyrethroid resistance phenotype, 55 survivors from permethrin exposure and the 19 dead females were genotyped for the presence of 1014F and 1014S *kdr* mutations. Genotyping was carried out using TaqMan real-time PCR thermocycler (Agilent Mx3005) following the protocols established by Bass and colleagues [28, 29]. 5 µl of Sensimix (Bioline), 0.25 µl of 40 × Probe Mix coupled to allelic-specific primers, 4.25 µl of dH_2_0, and 1 µl of genomic DNA were constituted in a total volume of 10 µl. Thermocycling conditions were initial 10 min at 95 °C, followed by 40 cycles each of 92 °C for 15 s, and 60 °C for 1 min. Two probes labelled with fluorochromes FAM and HEX were used to detect the mutant alleles and the wild type susceptible alleles, respectively. Genotypes were scored from scatter plots of results produced by the Mx3005 v4.10 software. Three positive samples of known genotypes: (i) homozygote resistant for 1014F or 1014S *kdr*; (ii) heterozygote for 1014F or 1014S *kdr*; and (iii) susceptible L1014 were added as positive controls for each of the two experiments. 1 µl of ddH_2_0 was added to the negative control well. The correlation between the *kdr* genotypes and permethrin resistance phenotype was calculated by estimating the odds ratio (OR) and the statistical significance based on Fisher’s exact probability test.

#### Assessment of genetic diversity in the kdr locus of the voltage-gated sodium channel (VGSC)

To assess the strength of selection pressure acting on the mosquito population in relation to insecticide resistance, the genetic diversity of a fragment spanning exon-20 of the IIS6 of the VGSC (starting from intron-1 of the IIS6 to intron-2) was amplified from 8 each of the permethrin-alive and dead females. DNA was extracted as described above and amplification carried out with primer pairs: *kdr*CL-F (5′-AAATGTCTCGCCCAAATCAG-3′) and *kdr*CL-R (5′-GCACCTGCAAAACAATGTCA-3′) described by Pinto [30]. To 12.5 μl of the 2× AccuStartII PCR SuperMix (QuantaBio, Beverly, Massachusetts) containing optimized concentrations of MgCl_2_ and dNTP mixes, 0.2 μM each of the forward and reverse primer was added, followed by 1 μl cDNA. 10.5 μl of dH_2_0 was added to produce a total volume of 25 μl. Amplification was carried out using the following conditions: initial denaturation of one cycle at 94 °C for 3 min; followed by 35 cycles each of 94 °C for 30 s (denaturation), 60 °C for 30 s (annealing), and extension at 72 °C for 1 min; and one cycle at 72 °C for 5 min (final elongation). PCR products were cleaned individually with QIAquick^®^ PCR Purification Kit (QIAGEN, Hilden, Germany) and sequenced on both strands using the above primers.

Polymorphisms were detected through manual examination of sequence traces using BioEdit version 7.2.3.0 [31] and CLC sequence viewer (QIAGEN, Hilden, Germany), whereas haplotype reconstruction and analyses of genetic parameters of polymorphism were done using the DnaSP 5.10 [32]. Different sequences were compared by constructing a phylogenetic maximum likelihood tree using MEGA 6.0 [33]. To estimate genealogies between sequences of the alive and the dead mosquitoes, haplotype network was created with the TCS (http://darwin.uvigo.es/software/tcs.html) and tscBU [34]. All DNA sequences from the alive and dead females were submitted to the GenBank (MK440265-440280).

### Statistical analyses

To calculate the odds ratio the epiR package of R version 3.5.0 (https://cran.r-project.org/bin/windows/base/) was utilized. Estimation of LT_50_ was also carried out with probit analyses (glm with MASS package of R). Result of mortalities from synergist-deltamethrin exposure was compared with that obtained from exposure to deltamethrin alone using a two-tailed Chi Square test of independence as implemented in GraphPad Prism 7.02 (GraphPad Inc., La Jolla, CA, USA) and correlation between *kdr* genotype and permethrin resistance phenotype determined using Fisher’s exact test.

## Results

### Mosquito species composition

Out of the 484 mosquitoes caught resting indoor, 445 were Anopheles [51♂, 394♀ (316 blood fed and 78 unfed)], 39 were of *Culex* species (27♀ blood fed and 12♂). All the *Anopheles* were established to be *An. coluzzii*. From the 316 female *An. coluzzii* transferred into 1.5 ml only 147 (46.5%) laid eggs successfully, due to high mortality experienced while travelling to insectary at Kano, Nigeria. A total of 117 mosquitoes were caught from PSC, 106 of them *An. coluzzii* [9♂, 97♀ (95 blood fed and 2 unfed)], and 11 were of *Culex* species (8♀ blood fed and 3♂).

### Malaria transmission parameters

#### Indoor-resting density and human blood index

Indoor resting density of the female *An. coluzzii* was calculated from the number of females collected as 12.125 females per house. DNA extracted from 95 blood fed females collected using PSC was analysed for blood source. 91 of the abdomens contained human blood, 2 abdomens contained cow blood, and the rest failed. The human blood index was found to be very high, calculated as 0.957 (~ 96% of the foraging female *An. coluzzii* have fed on humans).

#### Estimation of sporozoites infection rate

93 DNA-extracted heads/thoraces from PSC-caught *An. coluzzii* were used to establish infection with *Plasmodium*. 13 mosquitoes were infected with *Plasmodium*. 12 heads/thoraces contained *P. falciparum* (F + = 12), corresponding to a sporozoite rate of 12.9%, and a single sample was also found infected with *P. ovale*/*P. vivax*/*P. malariae* (OVM). Nested PCR confirmed this parasite as *P. vivax* (V + = 1.0).

### WHO insecticide susceptibility bioassays

**Fig. 2:**
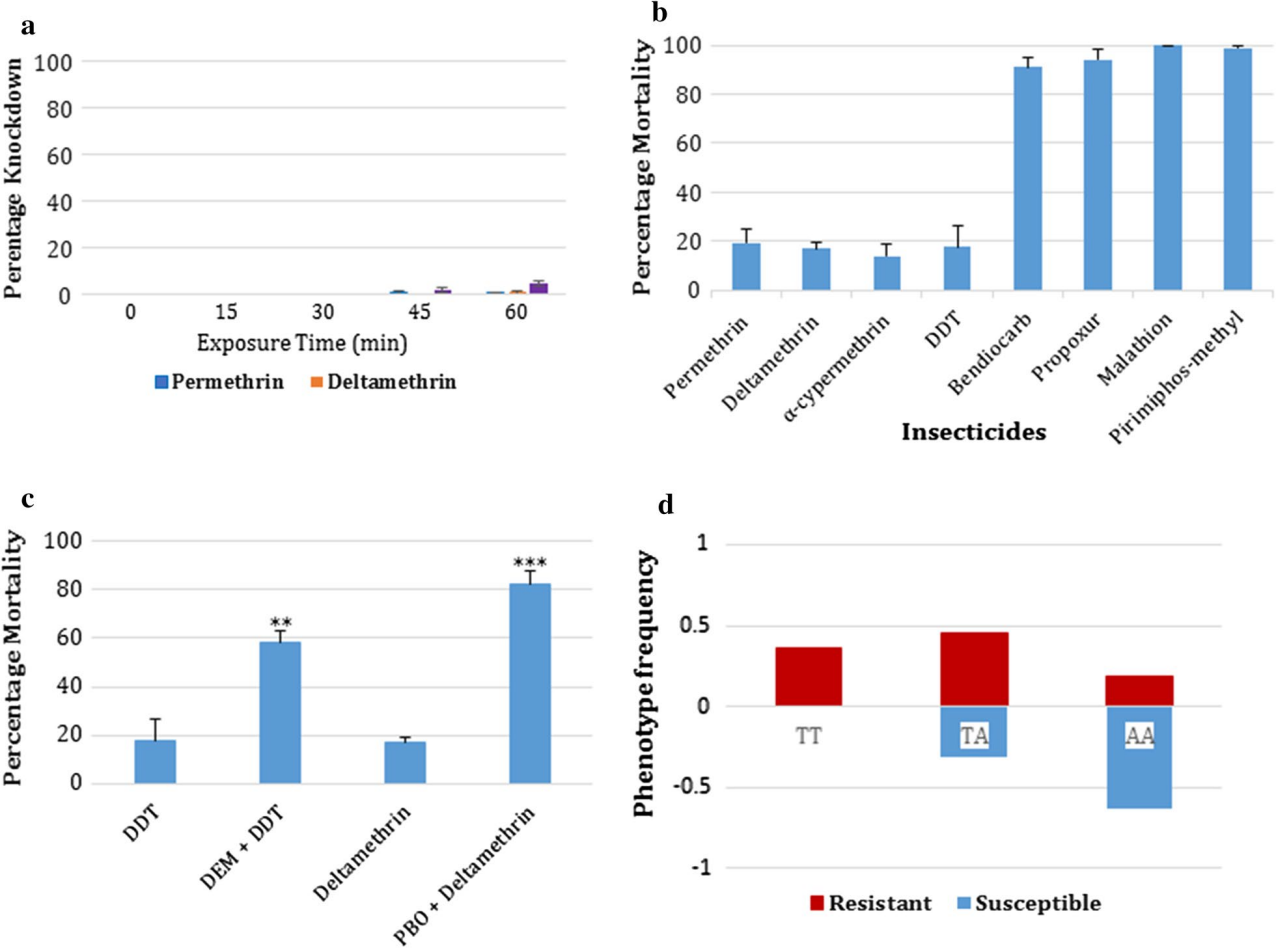
Resistance profiles of F_1_ Takatsaba *Anopheles coluzzii* females. **a** Knockdown resistance profile with permethrin, deltamethrin and DDT; **b** WHO bioassays with insecticides from different classes. **c** Effect of pre-exposure to synergists PBO against deltamethrin, and with DEM against DDT. Results shown are average of percentage mortalities from 4 replicates each ± SEM. No mortality was obtained in control females and positive control females exposed to DEM and PBO alone; ** and *** significantly different from exposure to DDT and deltamethrin alone, at p < 0.001 and p < 0.0001. **d** Correlation between the *kdr* genotype and resistance phenotypes in alive and dead Takatsaba *An. coluzzii*

The fully susceptible Ngoussou exhibited 100% mortalities against all insecticides tested. Bioassays with Takatsaba females revealed high resistance to pyrethroids and DDT with no knockdown at all for permethrin, deltamethrin and DDT in the first 30 min of exposure (Fig. 2a). At 45 min only a single mosquito was knocked down from permethrin exposure; and 2 from DDT. For DDT knockdown rate increased to only 5 individuals at 1 h, while only a single individual was knocked down for permethrin and deltamethrin, respectively at 1 h.

For the pyrethroids, mortalities of only 19% (95% CI 18–20), 17% (CI 16–18) and 14% (CI 9–19) were obtained at 24 h post-exposure, with permethrin, deltamethrin and α-cypermethrin, respectively (Fig. 2b). The same pattern was observed with DDT with only 18% (CI 16–19) of females dead at 24 h.

In contrast, the mosquitoes were more susceptible to the non-pyrethroid insecticides. Mortalities of more than 90% were observed for the carbamates bendiocarb and propoxur, while full susceptibility was seen with the organophosphates, malathion and pirimiphos-methyl (Fig. 2b).

### Estimation of resistance intensity

To establish levels of resistance WHO tube bioassays were conducted with deltamethrin varying exposure times from 60 to 300 min. The LT_50_ of Takatsaba *An. coluzzii* population was estimated as 156.65 min (95% CI 144.10–169.21, Fiducial). The LT_50_ for the Ngoussou has been estimated as 3.320 min (CI 2.67–3.97) from a previous study (Ibrahim et al. In Press, http://www.nature.com/articles/s41598-019-43634-4). The resistance ratio for Takatsaba females compared with the Ngoussou colony was thus calculated as 47.18.

### Investigating the potential role of metabolic resistance using synergist bioassays

Pre-exposure to the synergists recovered susceptibility to both deltamethrin and DDT, suggesting the possible role of cytochrome P450s and glutathione S-transferases respectively, in the resistance to the above insecticides. A high recovery of susceptibility was obtained following pre-exposure to PBO with mortality increasing ~ 5 times, from 17% seen in the conventional bioassay to 82% (CI 76–88) (Fig. 2c). Two-tailed test of independence indicated that the association between the mortality and PBO pre-exposure is highly significant (χ^2^ = 84.51, df = 1, p < 0.0001). DDT mortality increased also following pre-exposure to DEM, from 18% in the conventional bioassay to 58% (CI 52–65, χ^2^ = 33.96, df = 1, p < 0.001). No mortality was observed in all controls.

### Polymorphism analysis of the voltage-gated sodium channel

#### Investigation of the role of 1014F and 1014S kdr mutations on pyrethroid resistance

To determine correlation between the resistance phenotype and *kdr* genotype, 55 permethrin-resistant and 19 dead F_1_ females were genotyped for 1014F and 1014S *kdr* mutations. High frequency of 1014F *kdr* mutation was obtained with 20 females (36.36%) homozygote resistant (T/T) for 1014F *kdr* mutation (Table 1), and 25 females (45.45%) heterozygote resistant (T/A). Only 10 females (18.18%) harbour the homozygote susceptible allele (A/A). The 1014F *kdr* genotype frequency was established in the resistant females as ~ 0.82, and for all alive and dead females as 70.3%. Not surprisingly, out of the 19 permethrin-susceptible females successfully genotyped, 12 (63.15%) were homozygote susceptible at the 1014 codon (A/A); none were homozygote resistant; and 7 (36.84%) were heterozygotes (T/A). The *kdr* genotype frequency in the susceptible mosquitoes was established as 0.37. The 1014S *kdr* mutation was not detected in all mosquitoes genotyped.

**Table 1:** Correlation between the 1014F allele frequency and permethrin resistance phenotype in Takatsaba *Anopheles coluzzii* populations

Population	Phenotype	n	L1014F alleles			% *kdr* frequency (RR + RS)	*kdr* allele	Odds ratio (RR + RS vs SS)	F p value
			TTT (RR)	TTT/A (RS)	TTA (SS)				
Takatsaba♀	Alive	55	20 (36.36%)	25 (45.45%)	10 (18.18%)	45 (81.81%)	0.82	7.71 (2.43–14.53)	(0.001)
	Dead	19	0 (0%)	7 (36.84%)	12 (63.15%)	7 (36.84%)	0.37		
Total		74	20 (27.02%)	32 (43.24%)	22 (29.73%)	52 (70.27%)			

n, number of successfully genotyped individuals. Numbers in brackets represent percentage frequency. TTT: homozygote resistant alleles (RR); TTT/A: heterozygote resistant; and TTA: homozygote susceptible. F, Fisher’s exact test

A significant difference in genotype distribution was observed between the permethrin-resistant and susceptible individuals (Table 1, Fig. 2d), with 63% of susceptible females homozygote susceptible for 1014F *kdr*, while only 18% of the resistant females harbours the susceptible allele. A significant correlation was observed between permethrin resistance and presence of 1014F mutation [odds ratio of 7.71 (95% CI 2.43–14.53, χ^2^ = 13.67, p = 0.001)] (Table 1) when comparing the frequency of the resistance (homozygote resistant, RR + heterozygote resistant, RS) with susceptible allele (SS), in all females genotyped. However, stronger association was observed in the RR females in relation to the SS females [OR of 22 (CI 2.48–95.27, χ^2^ = 11.67, p = 0.001)] compared to above and to the association observed between the SS and the heterozygote females (RS) [OR of 4.29 (CI 1.31–14.03, χ^2^ = 6.10, p = 0.014)].

#### Genetic diversity of the IIS6 fragment of the voltage-gated sodium channel

**Fig. 3:**
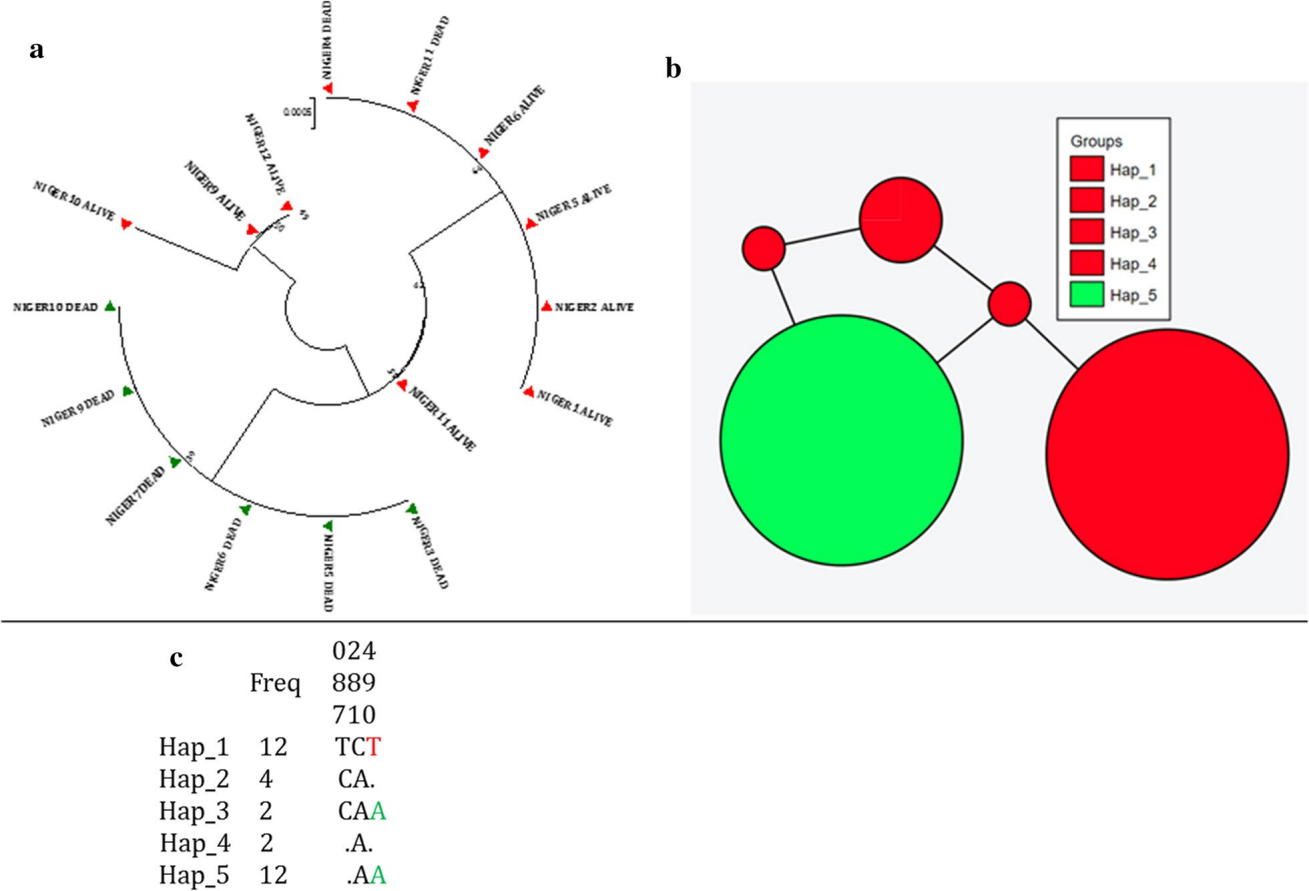
Pattern of genetic variability and polymorphism of the voltage-gated sodium channel in *Anopheles coluzzii*. **a** Maximum likelihood phylogenetic tree of intron-1 DNA sequences; **b** haplotype networks (TCS) for the fragment of the VGSC sequences in. Haplotypes are scaled accordingly to reflect their respective frequencies; **c** polymorphic positions of the VGSC fragment in alive and dead mosquitoes showing the haplotypes frequencies

A 545 bp sequence was successfully retrieved from sequencing data and analysed for genetic diversity. This fragment spanning exon-20 of the IIS6 of the VGSC from 8 each of alive and dead females revealed a higher heterogeneity within the alive mosquitoes compared to the dead ones. Six sequences each from the alive and dead formed two predominant clusters: Hap_1 and Hap_5 respectively (Fig. 3a–c) corresponding to the resistant allele 1014F (Hap_1) and to the susceptible allele L1014 (Hap_5). However, sequences from two dead mosquitoes cluster into Hap_1, resulting in 2 haplotypes for the dead mosquitoes, and lower haplotype diversity (Fig. 3a–c, Table 2), compared to the alive mosquitoes. Indeed, the alive mosquitoes contributed 4 haplotypes out of 5 with a diversity on average twice that of the dead mosquitoes, suggesting a potential segregation event taking place within intron-1 of the VGSC in the alive mosquitoes. Except for the 1014 codon, no other polymorphism (synonymous or nonsynonymous) was seen in the exon-20 in both alive and dead mosquitoes. Both the other two mutations were seen in intron-1, and none in intron-2.

**Table 2:** Summary statistics for polymorphism of the fragment of voltage-gated sodium channel haplotypes between alive and dead mosquitoes

Phenotype	N	S	h	H_d_	Syn	Nonsyn	π (k)	D (Tajima)	D* (Fu and Li)
Alive	8	3	4	0.70	0	1	0.00230 (1.266)	1.16^ns^	1.04^ns^
Dead	8	2	2	0.40	0	1	0.01470 (0.800)	0.84^ns^	0.91^ns^
All	16	3	5	0.72	0	1	0.0024 (1.306)	0.94^ns^	1.36^ns^

N, number of sequences (n); S, number of polymorphic sites; h, haplotype; H_d_, haplotype diversity; Syn, synonymous mutations; Nonsyn, non-synonymous mutations; π, nucleotide diversity (k = mean number of nucleotide differences); Tajima’s D and Fu and Li’s D statistics, ns, not significant

## Discussion

Failure to make significant progress in malaria control in high burden countries like Niger Republic is jeopardising the WHO’s Global Technical Strategy for Malaria 2016–2030. Control efforts in Niger, like other Sahelian zone countries are not enough, due in part to lack of reliable surveillance data to guide decision making by the national malaria control programmes. To facilitate evidence-based malaria control approaches and resistance management in Niger, we established the role of the major malaria vector *An. coluzzii* in malaria transmission in an area of stable transmission. Insecticide resistance profile of this malaria vector was established and the possible molecular mechanisms driving the resistance in the field investigated. However, conclusions of this study are drawn from a single collection, at one site. To capture the actual level/variation in *Plasmodium* infection and pattern of insecticide resistance in this malaria vector across southern Niger there is a need to carry out further collection at different time points, and several sites spanning the Sahel of the country.

Over the years, *An. coluzzii* has progressively become the DVS in the Sahel zone of Niger compared to the *Anopheles arabiensis* which is hard to come by, and *An. gambiae* sensu stricto, which has virtually disappeared in this region [9, 11]. Indeed, in recent years this species has been reported more than the other sibling species in various studies from the Sudan/Sahel of the neighbouring countries (e.g. in northern Nigeria [35], in northern Chad [36] and in Mali [37]), suggesting that this malaria vector has probably adapted well in the Sahel regions.

Also, *Plasmodium* transmission competence of *An. coluzzii* has increased in Niger with time. Previous studies have reported sporozoite rate of no more than 3% in various sites across Niger [10, 11]. The finding of *P. falciparum* sporozoite rate of ~ 13% in this study suggests that this vector is sustaining a high level of malaria transmission despite ongoing control efforts. This coupled with the high anthropophagic index of more than 95% suggests that this species has become a very efficient transmitter of malaria parasite in the Sahel and must be targeted to reduce malaria burden in this region. However, the high infection observed might also be because some of the females had recently taken blood from infected individuals.

The fact that mortalities of less than a quarter was recorded for the pyrethroids and DDT suggests that the resistance to these nerve agents have escalated in *An. coluzzii* from southern Niger. In comparison to the findings of Soumaila and colleagues [9] at Tessaoaua, resistance towards permethrin and α-cypermethrin has doubled and quadrupled, respectively in a few years. This is possibly due to increased selection pressure from second wave of distribution of PermaNet2.0 LLIN between 2014 and 2017. Also, in contrast to the previous observation of full susceptibility towards carbamates [9] marginal resistance was observed, especially towards bendiocarb. The level of bendiocarb resistance resembles recent observation in the Sahel of Nigeria where *An. coluzzii* populations exhibited comparable moderate resistance (Ibrahim et al. In Press, http://www.nature.com/articles/s41598-019-43634-4). However, as previously reported the mosquitoes were fully susceptible to the organophosphates. The high pyrethroid resistance observed in the Tessaoaua populations is evident in its high LT_50_ with deltamethrin, which is on average higher than the values described previously for resistant populations *of An. gambiae* from the Tororo (Uganda) and Tiefora (Burkina Faso) [38].

The high recovery of resistance on pre-exposure to PBO and DEM points to the potential role of P450 monooxygenases and glutathione S-transferases, respectively in the resistance. Previous studies have reported the use of synergists to investigate contribution of metabolic resistance in various *An. gambiae* populations [27, 39].

A previous study has reported the 1014F *kdr* frequency of 56% in *An. coluzzii* from Tessaoua [9]. This frequency has increased to ~ 82% in 5 years, like the observations in the neighbouring Sahel region of Nigeria [35]. The low haplotype and nucleotide diversity seen in the Niger populations agrees with the previous observation that Western African sites variation at the intron-1 was lowest compared to other regions of Africa [30]. The low genetic diversity (highlighted by the very low number of haplotypes) suggests a restricted polymorphism of the VGSC, in link with the near fixation of the 1014F allele in this population. These findings are consistent with previous studies reported as reported in West [35] and Central Africa [40].

## Conclusion

In a few decades *An. coluzzii* has adapted to the dry conditions of the Sahel region to become the major malaria vector (e.g. in Niger). Pyrethroid resistance in this species has escalated and will pose a threat to the malaria control/elimination agenda. Of significant importance also is the carbamate resistance observed in the field which could confound feature control measures using carbamate-based the indoor residual spraying. It is necessary to continue monitoring the spacio-temporal pattern of this resistance in the south of Niger and to carry out detailed studies on the contribution of metabolic resistance mechanisms operating in the field populations of this species from Niger. This will help revamp the effort towards malaria control in Niger/Sahelian region in general.

## Data Availability

DNA sequences reported in this paper were deposited at GenBank (Accession Number: MK440265-440280).
